# Peripheral Nerve Regeneration Using Different Germ Layer-Derived Adult Stem Cells in the Past Decade

**DOI:** 10.1155/2021/5586523

**Published:** 2021-09-09

**Authors:** Yu Li, Yuzuru Kamei, Miki Kambe, Katsumi Ebisawa, Mayumi Oishi, Keisuke Takanari

**Affiliations:** ^1^Department of Plastic and Reconstructive Surgery, Nagoya University Graduate School of Medicine, Japan; ^2^Department of Plastic and Reconstructive Surgery, Aichi Cancer Center, Japan

## Abstract

Peripheral nerve injuries (PNIs) are some of the most common types of traumatic lesions affecting the nervous system. Although the peripheral nervous system has a higher regenerative ability than the central nervous system, delayed treatment is associated with disturbances in both distal sensory and functional abilities. Over the past decades, adult stem cell-based therapies for peripheral nerve injuries have drawn attention from researchers. This is because various stem cells can promote regeneration after peripheral nerve injuries by differentiating into neural-line cells, secreting various neurotrophic factors, and regulating the activity of *in situ* Schwann cells (SCs). This article reviewed research from the past 10 years on the role of stem cells in the repair of PNIs. We concluded that adult stem cell-based therapies promote the regeneration of PNI in various ways.

## 1. Introduction

Peripheral nerve injuries (PNIs) are one of the most common types of traumatic lesions affecting the nervous system. They have an incidence of between 13 and 23 per 100,000 persons per year in developed countries [1], although it has a relatively higher impact in developing countries [2]. PNI usually involves partial or total loss of motor, sensory, and autonomic functions as well as neuropathic pain owing to the loss of structure and function of peripheral nerves from trauma, accidents, and other causes.

**Figure 1:**
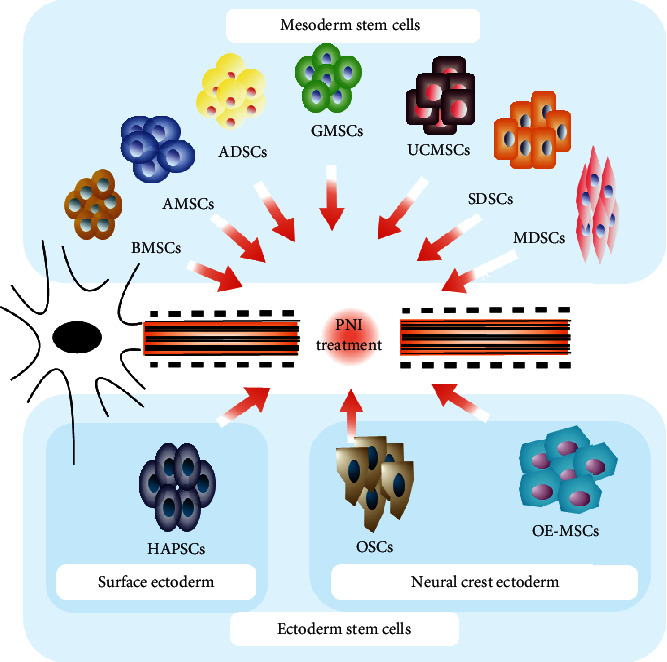
Adult stem cell types.

**Figure 2:**
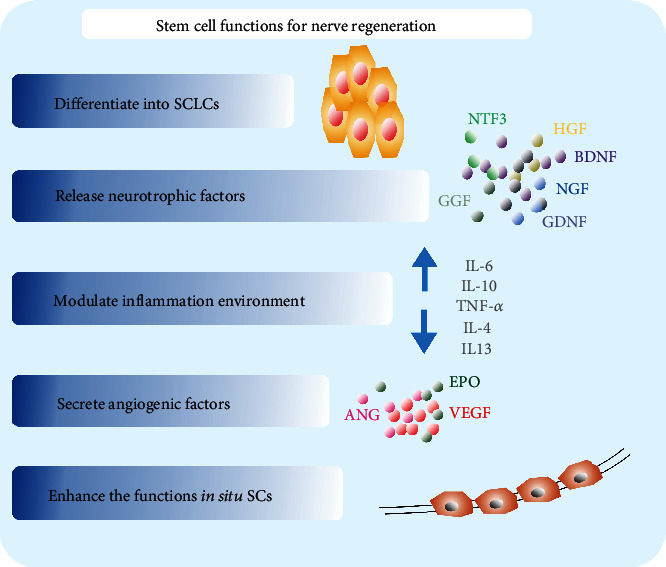
Functions of adult stem cells.

After PNI, a series of cellular and molecular events called Wallerian degeneration initially occurs. This is a process that clears debris from degeneration by degrading Schwann cells (SCs) and inducing the infiltration of microphages [3, 4]. Meanwhile, protein metabolism is altered, resulting in the activation of SCs [5]. These cells start forming structures known as “bands of Büngner” in order to provide guidance for axon regeneration. They also produce neurotrophic factors and extracellular matrix (ECM) molecules that promote axonal regeneration [6]. Therefore, SCs play a crucial role in peripheral nerve repair. Despite peripheral nerve axons possessing an intrinsic capability to generate and reconnect with their targets, in crucial nerve gaps, it is difficult to achieve complete functional and structural recovery [7]. This is because of the slow rate of axonal regeneration (1 mm/d) [8] as well as Wallerian degeneration occurring in the distal axon. Different therapeutic approaches have been investigated for handling lesions that cause large nerve gap, ranging from autologous nerve grafting, the gold standard treatment, up to various designed nerve guidance conduits (NGCs) combined with SC treatment [9]. The former is limited by poor functional outcomes, caused by scarce tissue graft availability and donor site morbidity. Furthermore, the latter is limited by difficulties in the harvesting and expansion of SCs. In this regard, researchers have begun to search for stem cells from different cell lineages, which are able to transform into SCs. We know that SCs are differentiated from the ectoderm, thus, this article reviewed the role and mechanisms of multifunctional adult stem cells from the ectoderm, as well as from the mesoderm and the neural crest of the fourth germ layer in peripheral nerve regeneration (Figures 1 and 2).

## 2. Adult Stem Cell-Based Therapies

Adult stem cells (ASCs), or somatic stem cells, are a collection of undifferentiated cells that are present in the postnatal body. Owing to their considerable self-renewal and multipotent ability, ASCs play an important role in PNIs. Recently, the beneficial effects of ASCs in the treatment of PNIs have been well studied, and comprehensive reports demonstrate their multilineage differentiation potential, secretion of neurotrophic factors, and immunomodulatory properties. Herein, we discuss the characteristics and functions of mesoderm-derived, surface ectoderm-derived, and fourth germ layer-derived stem cell types.

### 2.1. Mesodermal Cells: Mesenchymal Stem Cells

After a report in 1999 indicated that mesenchymal stem cells (MSCs) could be induced to transdifferentiate exclusively into adipocytic, chondrocytic, or osteocytic lineages [10], MSCs have been considered a hot topic in regenerative medicine. MSCs belong to the mesodermal lineage, but they are able to cross boundaries between mesodermal and ectodermal lineages, which include neural lineages [11]. Recently, MSCs are regarded as an important source of SCs because they are easily harvested from either the patient or donor-derived mesenchymal tissues, such as the bone marrow, adipose tissue, and umbilical cord. Furthermore, SCs are the main supportive cells for peripheral nerve regeneration; however, the SC buildup causes new damage to other peripheral nerve segments and also has several technical limitations regarding their cell-based therapy application. Therefore, MSCs are satisfactory candidates for use in PNI.

#### 2.1.1. Bone Marrow Mesenchymal Stem Cells

Bone marrow mesenchymal stem cells (BMSCs) have abundant sources and the potential to self-renew; however, they can also differentiate into several different lineages, including neuronal cell types such as SCs [12, 13]. Several studies have indicated that undifferentiated bone marrow mesenchymal stem cell (u-BMSC) transplantation promotes nerve regeneration in different animal models [14, 15]; however, some reports demonstrated that u-BMSCs failed to promote any significant changes in regeneration outcome [16, 17]. SC-like differentiated bone marrow stem cells (d-BMSCs) have been shown to be more effective at nerve functional and histological recovery [18, 19]. This suggests that d-BMSCs are functionally close to authentic SCs. The characteristics of the mechanisms underlying the BMSC regeneration enhancement are thought to be directly related to the release of axonal regeneration proteins such as growth-associated protein 43 (GAP-43) [20] and neurotrophic factors such as nerve growth factors (NGF) and brain-derived neurotrophic factors (BDNF) [21]. These exert immunomodulatory effects by changing the inflammatory environment [22, 23], while protecting the injured nerve area from fibrous tissue infiltration [24]. Other research groups also reported vital improvements in the regeneration of injured peripheral nerves following the transplantation of neurotrophic factor-transfected BMSCs, when compared to normal BMSCs. Therefore, gene-based cell therapies have attracted the attention of many scientists. Furthermore, Chen et al. [25] reported that the Hippo, Wnt, transforming growth factor-beta, and hedgehog signaling pathways are potentially associated with BMSC neural differentiation. Additionally, overexpression of microRNA-124 promotes the neuronal differentiation of BMSCs.

#### 2.1.2. Adipose-Derived Mesenchymal Stem Cells

Within the past decade, adipose-derived mesenchymal stem cells (ADSCs) have attracted the attention of researchers and clinicians in PNI repair. This is because they have the ability to differentiate into Schwan cell-like cells (SCLCs), downregulating inflammation [26]. Furthermore, they induce the direct effect of paracrine growth factors including NGF, BDNF, vascular endothelial growth factor (VEGF), hepatocyte growth factor (HGF), and insulin-like growth factor (IGF) [27], which indirectly affect endogenous SC activity [28–30]. It has been previously shown that ADSC-derived exosomes promote peripheral nerve regeneration by providing NGFs, optimizing the function of SCs *in situ* and reducing SC apoptosis *in vitro* and *in vivo* [25]. Regarding these functions, many experimental studies have been performed to demonstrate the beneficial effects of ADSCs (Table 1). Nevertheless, accumulating evidence indicates that differentiated ADSCs (dADSCs) show better axonal regeneration and functional recovery results than for undifferentiated-ADSCs (uADSCs) [31]. Several studies have reported that dADSCs express higher levels of neurotrophic factors, including NFG, BNDNF, GDNF, and NT4 [32–35], angiogenic factor (VEGF1), and ECM related protein (COL3A1) [36, 37] when compared with uADSCs, thus, accelerating axonal regeneration. Experimental studies in rat nerve injury models have investigated the effects of dADSCs (Table 2). However, because the dADSCs rapidly dedifferentiate in the absence of a stimulating medium [38], the differentiation process and method of cell transplantation to an injured environment must be studied prior to the use of dADSCs in PNI therapies. Thus, researchers applied ECM scaffolds containing fibronectin or laminin to create a similar microenvironment in order to maintain SC-like features for cell survival [39]. Interestingly, there was no significant difference between ADSCs and BMSCs when used in rat sciatic nerve injury. Both showed satisfactory results in terms of histological and functional recovery [40]. Furthermore, another study indicated that ADSCs successfully reduced neuropathic pain in the PNI model when compared with the BMSC group [41]. Recently, overexpressed neurotrophic factor ADSCs, via lentivirus transfection, became a new method to enhance PNI repair [42]. Without the donor site morbidity limitations associated with the isolation of SCs or BMSCs, ADSCs may provide a more effective cell population, thus, translating into the clinic to enhance PNI repair methods.

#### 2.1.3. Gingiva-Derived Mesenchymal Stem Cells

As an alternative and readily accessible source of stem cells for the repair of nerve tissues, gingiva-derived mesenchymal stem cells (GMSCs) have attracted the attention of researchers in recent years. GMSCs can be directly induced to become multipotent and expandable neural progenitor-like cell (NPCs) through nongenetic approaches [43, 44]. NPCs displayed better therapeutic effects on peripheral nerve regeneration than their parental GMSC counterparts. A study demonstrated that GMSCs and induced-NPCs promote peripheral nerve repair by promoting remyelination regulators, c-JUN, and Krox-20/EGR2 [44]. Furthermore, the same group suggested that the Yes-associated protein (YAP) signaling plays an important role in orchestrating the induction of NPCs from GMSCs [45]. Other researchers have used exosomes from the supernatant of cultured GMSCs for the treatment of PNI. Their results indicated that GMSC-derived vesicles or exosomes preferentially promote SC dedifferentiation and have the potential to activate the repair phenotype of SCs by regulating the expression of key transcription factors [46, 47]. These findings suggest that the practical advantages of GMSCs make them applicable for peripheral nerve injuries.

#### 2.1.4. Skin-Derived Stem Cells

Skin-derived stem cells (SDSCs) are an accessible source of multipotent stem cells extracted from the dermis. These cells were reported to generate both endothelial and neural derivatives *in vitro* and *in vivo* [48, 49]. Furthermore, research has shown that SDSCs can differentiate into SDSC-SCs by 95% *in vitro* as well as survive, migrate, and maintain the expression of SC markers, over long-term periods, when transplanted into acellular isografts *in vivo* [50]. Several studies have reported that SDSC-SC treatment provides PNIs with immediate axon regeneration, myelination, and functional recovery. They do this via secreting neurotrophic factors and promoting proliferation of denervated host SCs or recruiting them to the sites of injury [51]. Moreover, studies have suggested the beneficial effects of SDSC-SCs in delayed PNI and in enhancing muscle reinnervation [52, 53]. Regarding immunomodulatory function, Stratton et al. [54] transplanted SDSC-SCs into nerve-injury site. The results indicated that SDSC-SCs enhanced debris clearance and inflammation following injury by secreting cytokines such as IL-6. Moreover, another group proved that advanced debris clearance implies a less inhibitory microenvironment, which in turn contributes to axon regeneration [53]. Additionally, treatment of the SDSC-SCs sensory neurons [55] or motoneurons [52] could be significantly improved in PNI animal models. In a clinical environment, patient-derived SDSCs alongside a transplanted collagen artificial nerve graft promoted motor and sensory functions of the median nerve during the case follow-up period [56]. In addition, Brandenburger and Kruse's group [57] described a protocol in which a coculture system of peripheral nerve cells with sweat gland-derived stem cells promoted neurite outgrowth. Given their ease of accessibility, ability to differentiate, and their capacity to enhance axon regeneration, SDSCs are a strong candidate for therapeutic PNI.

#### 2.1.5. Muscle-Derived Mesenchymal Stem Cells

Under certain conditions, muscle-derived mesenchymal stem cells (MDSCs) can not only differentiate into mesoderm cells, including myocytes, adipocytes [58], and cartilage [59], but they can also differentiate into ectoderm cells such as neurocytes [60, 61] *in vitro*. Moreover, *in vivo*, some studies indicated that MDSCs could ameliorate critical-sized sciatic nerve injury in a murine model by differentiating into myelin-producing SCs [62, 63]. Meanwhile, Kazuno et al. [64] transplanted MDSCs into bioabsorbable polyglyconate (PGA) in a recurrent laryngeal nerve (RLN) transected mouse model and described good recovery of the RLN. Furthermore, Tamaki et al.'s group also suggested that MDSCs preferentially differentiate into perineurial/endoneurial cells, as well as SCs, *in vivo* [60]. They further sorted MDSCs into CD34(-)/CD45(-)/CD29(+) (Sk-DN/29(+)) and CD34(+)/CD45(-) (Sk-34) cells. This was followed by their expansion and then cotransplantation; it was reported that the latter type of MDSCs can differentiate into vascular endothelial cells and pericytes *in vivo* [65], which significantly improved functional recovery by promoting axon growth and vascular formation [66]. Furthermore, other studies also transplanted MDSCs with overexpressed the neurotrophic factor and achieved high-quality PNI healing [67]. Thus far, the main problem is that more reliable MDSC expansion protocols are needed for future clinical applications.

#### 2.1.6. Amniotic-Derived Mesenchymal Stem Cells

Amniotic-derived mesenchymal stem cells (AMSCs) are a kind of multipotent stem cell with the capabilities of MSCs [68]. During the early years, intravenous administration of AMSCs provided beneficial effect on PNI because they express stromal cell-derived factor-1*α* (SDF-*α*) and its receptor chemokine receptor type-4 (CXCR-4) to enhance nerve regeneration by recruiting progenitor cells [69]. And another study also showed the therapy could alleviate the neuropathic pain and suppress the inflammation response by expressing IL-1*β*, TNF-*α* [70]. Furthermore, AMSCs produce a number of neurotrophic factors such as GAP-43, NGF, BDNF, and GDNF, which promote the nerve injury recovery in different animal models [71]. Additionally, Li et al.'s group indicated that AMSC transplantation display neurovascular tropism, which could aid in the recovery of sciatic nerve injury [72]. Besides, when compared to traditional BMSCs, AMSCs showed higher proliferative capacity and more efficiency nerve growth factor secretion both in vivo and vitro [73]. Therefore, AMSCs are a promising alternation for therapy of PNI.

#### 2.1.7. Umbilical Cord-Derived Mesenchymal Stem Cells

Umbilical cord-derived mesenchymal stem cells (UCMSCs) are able to give rise to multiple cell types of neural lineage both in vivo and vitro [74]. Recent studies have showed that UCMSCs can be effectively used for peripheral nerve regeneration due to its paracrine [75], immunomodulatory, antioxidative [76], as well as inflammation modulatory characteristics [77]. Those properties could supply a favorable microenvironment for nerve regeneration. Furthermore, when combined with the biomaterial nerve conduit, UCMSCs exhibit beneficial effect in PNI. Cui et al. utilized the collagen conduit loaded with UCMSCs to the sciatic nerve defect in dogs and suggested that the therapy improved functional and histological recovery [78]. Another study indicated that associating a hybrid chitosan membrane with UCMSCs enhanced the motor and sensory functional recovery in rat model by stimulating the UCMSC differentiation in to SCLCs [79]. With regarding clinical application, one study reported radial nerve injury patients treated with UCMSC-loaded amniotic membrane displayed obvious improvement in muscular strength after 12 weeks when compared with control patients [80]. However, even though the UCMSCs are easy to harvest and purify, the main disadvantage is that it is hard to collect enough UCMSCs to transplant an adult.

### 2.2. Ectodermal Cells

The fact that embryonic origin is shared with the peripheral nervous system allows us to argue that ectodermal cells are much closer to nerve cells than mesodermal MSCs. In this section, two types of ectodermal cells, the surface ectoderm and neural crest ectoderm, are discussed. The latter is regarded as the fourth germ layer because of its multipotency, long-range migration throughout the embryo, and its capacity to generate a prodigious number of differentiated cell types. In the following subsections, we review the contribution of ectodermal cells originating from various adult tissue types for PNI regeneration.

#### 2.2.1. Hair Follicle-Associated-Pluripotent Stem Cells

Hair follicle-associated pluripotent stem cells (HAPSCs) are a typical source of surface ectodermal cells. In 2004, researchers showed that neural crest cells (NCCs) grew out when the hair follicle bulge was explanted, resulting in differentiation into a variety of cell types, including neurons, smooth muscle cells, rare SCs, and melanocytes [81]. Furthermore, Amoh et al. (2005, 2010, 2012) proved that HAPSCs could differentiate into glial fibrillary acidic protein-positive SCs *in vivo* and form a myelin sheath surrounding axons while the severed sciatic nerve regrew in mice [82, 83]. Furthermore, this same group implanted mouse green fluorescent protein- (GFP-) expressing HAPSC spheres, encapsulated in polyvinylidene fluoride- (PVDF-) membrane cylinders, into the severed sciatic nerve of immunocompetent and immunocompromised (nude) mice. Eight weeks after treatment, the transplanted group showed greater improvement both in hematoxylin and eosin (H&E) staining and quantitative walking analysis than with the transplantation of empty cylinders. These findings suggest that HAPSCs provide a potentially accessible, autologous source of stem cells for PNI regeneration therapy.

#### 2.2.2. Olfactory Stem Cells

Olfactory stem cells (OSCs) are a type of neural crest cell in mammals, including humans. During the past decade, OSCs have attracted considerable interest due to their self-renewal ability, as well as their ability to express different glial markers [84] and myelin constituents in adult mammals [85], thereby providing paracrine factors and a favorable microenvironment for neurogenesis [86]. Their therapeutic potential has been successfully tested in various PNI animal models (Table 3). One study confirmed that stem cells from the olfactory mucosa produce various growth factors and cytokines such as Galectin-1, growth arrest-specific 1 (GAS1), insulin-like growth factor-binding proteins 2 and 3 (IGF-BP2 and IGF-BP3), soluble tumor necrosis factor receptor I (sTNF-RI), and tumor necrosis factor-related weak inducer of apoptosis receptor (TWEAKR), all of which accelerate functional recovery after facial nerve injury in mice [87]. Additionally, when combined with biodegradation nerve conduits, olfactory ectomesenchymal stem cells (OE-MSCs) are also regarded as a good option. Salehi et al. (2019) utilized an alginate/chitosan hydrogel saturated with OSCs to treat sciatic nerve defects. They found that the therapy enhanced regeneration when compared to both the control and hydrogel without cells groups [88]. Furthermore, OSCs can be easily obtained from olfactory mucosa biopsies with limited risk, making it possible to envisage autologous cell transplantation strategies in future clinical work.

#### 2.2.3. Dental Ectomesenchymal Stem Cells

Dental ectomesenchymal stem cells (DE-MSCs), which originate from most craniofacial structures, such as the dental pulp, periodontal ligament, and exfoliated deciduous teeth, have the same origin as neural crest cells [89]. Over the past decade, dental pulp stem cells (DPSCs) have been confirmed to be capable of differentiating into functional oligodendrocytes *in vitro* [90], which highlights their potential application in PNI repair. Previous findings have shown that DPSCs promote axonal regeneration by releasing growth factors, including BDNF, GDNF, NGF, NTF3, ANGPT1, and VEGFA [91]. Furthermore, Sanen et al. [92] applied differentiated DPSCs in a rat sciatic nerve injury model and reported the proangiogenic effects of differentiated human DPSCs (d-hDPSCs) in PNI treatment. Recently, several investigations have also proven that DPSCs combined with different artificial nerves favor the peripheral nervous system [93]. They highlighted that both customized 3D nanofibrous scaffolds and chitosan-scaffolds support proliferation and neural differentiation of DPSCs, thus, they could be utilized for PNI repair. A few studies have also described the paracrine activity and differentiation potential of stem cells from exfoliated deciduous teeth (SHED) and periodontal ligaments (PDLSCs) [94]. Data from different studies (Table 4) suggest that these DE-MSCs not only show pluripotent differentiation potential but also have promising regenerative potential.

### 2.3. Endodermal Cell

Primary cells from endodermal organs such as the liver, lung, pancreas, and digestive tract are often difficult to grow *in vitro*, while the procurement of primary tissue is often ethically questionable, especially from healthy donors. Thus, nerve regenerative medicine applications are currently limited by the lack of high-quality endodermal adult stem cells.

## 3. Conclusions and Future Perspectives

Due to their multipotential, paracrine, and ethically friendly properties, ASC therapies are gradually garnering more attention as an efficient solution to healing PNIs. In this review, we sorted ASCs based on the three germ layers. Ideally, in this application, the ASCs should originate from mesodermal tissue because they not only retain the characteristics of mesodermal cells but can also differentiate into neural lineage cells, which are beneficial for peripheral regeneration. In addition to MSCs, ectoderm-derived stem cells also enable nerve regeneration in preclinical treatments. In particular, neural crest stem cells (NCDSs), which have the same germ layer as neurocytes, can express nerve-specific markers. Furthermore, with their multipotent ability, NCSCs expedite the development of ectodermal stem cell-based therapies to treat PNI. Over the past decade, combining ASCs with tissue-engineered nerve conduits has accelerated the therapeutic effects of peripheral nerve repair.

Despite great promise in ASCs, some issues still exist that affect the efficiency of ASC-based therapy (Table 5). For instance, when ASCs are transplanted into PNI animal models, the percentage of ASCs differentiating directly into supportive cells and the ratio of surviving cells should be noted. Furthermore, some data support that differentiated cells rapidly dedifferentiated when there is a lack of stimulation [38]. Thus, the stimulator, mobilization, homing, and delivery system should be taken into consideration to improve the quantity and quality of stem cells in therapeutic environments.

## Figures and Tables

**Table 1:** Effect of ADSCs in PNI animal models.

Stem cell characteristics	Animal/nerve	Experimental model	Delivery system	Contribution to PNI regeneration	Reference
ADSCs	Mice sciatic nerve	5 mm nerve gap	Gelatin hydrogel tube	Promoted formation of myelin, restoration of denervation muscle atrophy	[95]
hADSCs	Rabbit peroneal	40 mm nerve gap	Vein conduit refilled with fibrin	Promoted myelination and axonal regeneration	[96]
ADSCs	Rat sciatic nerve	Nerve crush model	Perineural transplantation by cell injection	Gained better motor functional recovery and early proregeneration	[97]
ADSCs	Mouse sciatic nerve	Nerve crush model	Intravenous administration of ADSCs	Accelerated the functional recovery and reduced inflammatory infiltrates	[26]
ADSCs	Dog facial nerve	7 mm nerve gap	Core-Tex tube filled alginate hydrogel	Promoted nerve diameter, nerve number, and electrophysiological recovery	[39]
ADSCs	Rat sciatic nerve	10 mm nerve gap	Collagen conduit filled with fibrin-agarose hydrogels	Enhanced sensory and motor functional recovery	[98]
ADSCs	Rat sciatic nerve	10 mm nerve gap	Silicon rubber conduit	Augmented the functional recovery and axonal regeneration	[99]
ADSCs	Rat sciatic nerve	10 mm nerve gap	GGT (genipin-gelatin-tricalcium phosphate) nerve conduit	Promoted SFI and CMAPs (compound muscle action potentials)	[100]
ADSCs	Rat sciatic nerve	10 mm nerve gap	Acellular nerve tube	Increased walking behavior, nerve conduct velocity, myelinated fiber density	[101]
ADSCs	Rat sciatic nerve	10 mm nerve gap	Fibrin conduit	Enhanced axonal regeneration and angiogenesis	[36]
ADSCs	Rat sciatic nerve	10 mm nerve gap	3D-engineering of cellularized conduits	Augmented functional and histological assessment	[102]
ADSCs	Rabbit sciatic nerve	20 mm nerve gap	Acellular allogenic nerve	Improved recovery of nerve function, morphology, and tensile mechanical properties.	[103]
ADSCs	Rat sciatic nerve	Nerve transection	Fibrin glue	Enhanced the process of nerve regeneration and angiogenesis	[104]
ADSCs	Rat sciatic nerve	Nerve transection	Fibrin-hydrogel nerve conduits (FNC)	Promoted early nerve regeneration	[105]

**Table 2:** Effect of dADSCs in PNI animal models.

Stem cell characteristics	Cell markers	Animal/nerve	Experimental model	Delivery system	Contribution to PNI regeneration	Reference
Neuronally differentiated ADSCs	*β*III-tubulin	Rat sciatic nerve	10 mm nerve gap	Aligned PBHV nanofiber nerve scaffold	Improved motor functional and histological recovery	[106]
ADSCs-SC-like cells	GFAP, S100	Rat sciatic nerve	10 mm nerve gap	Acellular nerve allograft	Enhanced walking-track and electrophysiological result	[42]
Differentiated ADSCs	GFAP, S100	Rat sciatic nerve	15 mm nerve gap	Acellular nerve	Augmented histological and electrophysiological recovery	[107]
ADSCs-SC-like cells	S100, NGFR p75	Rat sciatic nerve	10 mm nerve gap	Biodegradable chitin conduit	Promoted motor functional and histological recovery	[108]
Differentiated ADSCs	S100, GFAP, p75	Rat sciatic nerve injury	15 mm nerve gap	NeuraWrap™ filled with EngNT-dADSC sheets	Supported neuronal regeneration in regard to myelination thickness and number of axons	[109]
Differentiated ADSCs	GFAP, S100	Rat sciatic nerve injury	15 mm nerve gap	PGA-c tube	Improved myelin formation and functional recovery	[110]
Differentiated ADSCs	GFAP, S100	Rat sciatic nerve injury	10 mm nerve gap	GGT nerve conduit	Promoted SFI, electrophysiological recovery, and gained equally result with autologous in histological analysis	[111]
Differentiated ADSCs	GFAP, *β*III-tubulin	Rat facial nerve	8 mm	Decellularized allogeneic artery conduits	Promoted nerve regeneration and functional restoration.	[112]
Differentiated ADSCs	GFAP, S100, p75	Rat sciatic nerve injury	10 mm nerve gap	PHB tube filled with fibrin glue	Increased axon myelination and functional recovery	[113]

**Table 3:** Effect of OSCs in PNI animal models.

OSC origin	Surface marker expression *in vitro*	Models of PNI	Delivery system	Contribution to PNI regeneration	Reference
hOE-MSCs	CD13, CD44, CD90, CD166, CD146, CD73, CD29, CD105	2 mm facial nerve gap in rats	Nerve stumps	Promoted the movement score and electrophysiological results	[114]
OE-MSCs	Nestin	20 mm inferior laryngeal nerve gap in rats	Cells are injected into nerve graft	Enhanced laryngeal mobility and function score	[115]
OE-MSCs	CD73, CD90, CD105, nestin, vimentin	Sciatic nerve crush in rats	Alginate/chitosan hydrogel	Improved motor and sensory nerve regeneration	[88]
OSCs	*β*-tubulin, nestin, and GFAP	10 mm sciatic nerve gap in rats	Biphasic conduit	Contributed functional, electrophysiological, and histological recovery	[116]
OSCs	*β*-tubulin, GFAP, nestin, OMP, Musashi-1, sox-2, Nanog	Facial nerve crush model in mice	Biodegradable hydrogel	Accelerated the recovery from facial palsy and enhanced nerve regeneration	[87]

**Table 4:** Effect of different ED-MSCs types in PNI animal models.

ED-MSC type	Surface marker expression *in vitro*	Models of PNI	Delivery system	Contribution to PNI regeneration	Reference
hDPSCs	P75NTR, GFAP, S100b, nestin, SOX-10, STRO-1, c-Kit, CD34	6 mm nerve gap in a rat sciatic nerve model	Collagen scaffold	Differentiated into Schwan cells *in vitro*, promoted myelin formation and functional recovery *in vivo*	[91]
DPSCs, PDLSC	CD73, CD90, CD105, CD146	10 mm nerve gap in a rat sciatic nerve model	Fibrin glue conduit	Beneficial effects on neurite outgrowth *in vitro*. Enhanced axonal regeneration *in vivo*	[117]
d-DPSCs	-	15 mm nerve gap in a rat sciatic nerve model	EngNT	Showed angiogenic properties *in vitro* and positive effect in nerve regeneration *in vivo*	[92]
DPSCs	-	7 mm nerve gap in a rat facial nerve model	PLGA tube	Promoted histological recovery *in vivo*	[118]
SHED	CD73, CD90, CD105, nestin, doublecortin, *β*-III-tubulin, NeuN, GFAP, S-100, A2B5, CNPase	12 mm nerve gap in a rat sciatic nerve model	Silicon conduit	Stimulated angiogenesis and neurite growth *in vitro*, enhanced functional and histological recovery *in vivo*	[94]
SHED	CD29, CD73, CD90, CD105, CD166, S100	5 mm nerve gap in a rat facial nerve model	PGAt nerve tube	Enhanced axonal regeneration and functional recovery	[119]
PDLSCs	POU4F2	Rat optic nerve crush model	Cells were injected into the vitreous chamber	Promoted neurite growth *in vitro*, improved optic nerve regeneration *in vivo*.	[120]

**Table 5:** Summary of pros and cons for stem cells.

		Pros	Cons	Reference
Mesodermal cells	BMSCs	(i) Self-renew ability (ii) Differentiate into neural lineage (iii) Produce neurotrophic factors (iv) Immunomodulatory effects	(i) Require invasive surgical procedures (ii) Donor site morbidity	[15, 18–21, 23, 121–126]
	ADSCs	(i) Self-renew ability (ii) Differentiate into neural lineage (iii) Produce neurotrophic factors (iv) Downregulating inflammation (v) Abundant source	(i) Require invasive surgical procedures (ii) Rapidly dedifferentiated	[25, 36, 38, 95, 103, 110, 127–132]
	GMSCs	(i) Self-renew ability (ii) Differentiate into neural lineage (iii) Promote *situ* SC dedifferentiation (iv) Phenotypically stable paracrine ability (v) Less invasive procedure	(i) Require invasive surgical procedures	[43–47]
	SDSCs	(i) Self-renew ability (ii) Differentiate into neural lineage (iii) Promote *situ* SC dedifferentiation (iv) Immunomodulatory effects	(i) Require invasive surgical procedures (ii) Low efficiency of isolation	[48, 49, 52–54, 56, 133–137]
	MDSCs	(i) Self-renew ability (ii) Differentiate into neural lineage (iii) Promote vascular formation (iv) Produce neurotrophic factors	(i) Require invasive surgical procedures (ii) Donor site morbidity (iii) Low efficiency of expansion	[60, 62, 63, 65, 67, 138]
	AMSCs	(i) Self-renew ability (ii) Differentiate into neural lineage (iii) Produce neurotrophic factors (iv) Promote vascular formation (v) Downregulating inflammation	(i) Difficult to isolation (ii) Unstable phenotype	[73, 139–144]
	UCMSCs	(i) Self-renew ability (ii) Differentiate into neural lineage (iii) Produce neurotrophic factors (iv) Downregulating inflammation (v) Easy to harvest and purify	(i) Risk of tumorgenesis (ii) Not abundance	[2, 75, 76, 78, 145–153]
Ectodermal cells	HAPSCs	(i) Self-renew ability (ii) Differentiate into neural lineage (iii) Readily accessible (iv) Abundance	(i) Unclear mechanism and condition for differentiation	[5, 81–83, 154, 155]
	OSCs	(i) Self-renew ability (ii) Differentiate into neural lineage (iii) Produce neurotrophic factors (iv) Readily accessible (v) Abundance	(i) Limited migration ability under hypoxic environment at the site of injury	[86, 87, 88, 156]
	DE-MSCs	(i) Self-renew ability (ii) Differentiate into neural lineage (iii) Produce neurotrophic factors (iv) Ease isolation procedure	(i) Low yield of stem cells	[89, 92–94, 157]
